# Application of Bacteriophage-containing Aerosol against Nosocomial Transmission of Carbapenem-Resistant *Acinetobacter baumannii* in an Intensive Care Unit

**DOI:** 10.1371/journal.pone.0168380

**Published:** 2016-12-16

**Authors:** Yu-Huai Ho, Chun-Chieh Tseng, Lih-Shinn Wang, Yi-Ting Chen, Guan-Jin Ho, Teng-Yi Lin, Ling-Yi Wang, Li-Kuang Chen

**Affiliations:** 1 Division of Infectious Diseases, Department of Internal Medicine, Buddhist Tzu Chi General Hospital and Tzu Chi University, Hualien, Taiwan; 2 Department and Graduate Institute of Public Health, Tzu Chi University, Hualien, Taiwan; 3 Medical Intensive Care Unit, Department of Internal Medicine, Buddhist Tzu Chi General Hospital and Tzu Chi University, Hualien, Taiwan; 4 Department of Surgical Critical Care Unit, Buddhist Tzu Chi General Hospital and Tzu Chi University, Hualien, Taiwan; 5 Department of Laboratory Medicine, Buddhist Tzu Chi General Hospital, Hualien, Taiwan; 6 Department of Medical Research, Buddhist Tzu Chi General Hospital, Hualien, Taiwan; 7 Institute of Medical Sciences, Department of Laboratory Diagnostic, College of Medicine, Tzu Chi University, Hualien, Taiwan; 8 Department of Laboratory Medicine, Clinical Pathology, Buddhist Tzu Chi General Hospital, Hualien, Taiwan; Universitatsklinikum Munster, GERMANY

## Abstract

**Background:**

Carbapenem-resistant *Acinetobacter baumannii* (CRAB) is associated with nosocomial infections worldwide. Here, we used phage as a potential agent to evaluate the efficacy of daily cleaning practices combined with a bacteriophage-containing aerosol against CRAB.

**Methods:**

A two-phase prospective intervention study was performed at a 945-bed public teaching hospital. From March to December 2013, we performed terminal cleaning using standard procedures plus an aerosol with active bacteriophage in the intensive care units to evaluate the impact on nosocomial incidence density, carbapenem-resistance rates and antimicrobial drug consumption amounts. Patients with culture proven CRAB infection were transferred to the isolation room when the phage aerosol cleaning had been completed.

**Results:**

A total of 264 new acquisitions of CRAB were identified in the intensive care units (191 in the pre-intervention period and 73 in the intervention period). The rates of new acquisitions of CRAB in the intensive care units decreased from 8.57 per 1000 patient-days in the pre-intervention period to 5.11 per 1000 patient-days in the intervention period (*p* = 0.0029). The mean percentage of resistant isolates CRAB decreased from 87.76% to 46.07% in the intensive care units (*p* = 0.001). All of the antimicrobials showed a significant reduction in consumption except imipenem.

**Conclusions:**

The bacteriophage was successful in decreasing the rates of infection caused by CRAB across intensive care units in a large teaching hospital.

## Introduction

*Acinetobacter baumannii* has emerged as an important nosocomial pathogen. According to the Centers for Disease Control, Department of Health, Taiwan, *A*. *baumannii* is one of the top three pathogens responsible for healthcare-associated infections in intensive care units (ICUs) in Taiwan. The proportion of carbapenem-resistant *A*. *baumannii* (CRAB) in medical center ICUs has increased from 16.4% in 2003 to 71.2% in 2012 [1, 2]. Infection caused by this organism has been associated with increased patient morbidity, length of hospital stay, and health care costs [3–5]. The crude mortality rate for nosocomial bloodstream infections caused by *A*. *baumannii* was 34% in a large US study but increased to 43% for bloodstream infections involving patients in ICUs [6]. Therefore, it is a serious public health problem because of the higher mortality and morbidity due to nosocomial CRAB bloodstream infection and there are limited choices for appropriate antimicrobial therapy [7].

To prevent the rapid spread of CRAB, it is important to educate all healthcare workers regarding appropriate infection control measures, including strict hand hygiene, judicious antimicrobial use, implementation of contact precautions for all patients known to be colonized or infected with target multiple-drug resistant organisms, and proper environmental decontamination [8]. To date, several disinfection techniques have been evaluated for inactivation of *A*. *baumannii* including pasteurization, ultraviolet light, chemical sanitizers, hydrogen peroxide, and photocatalysis. However, in these cases, terminal cleaning may not have been conducted thoroughly and the room surface may have remained contaminated with *A*. *baumannii* at levels as high as 60% after terminal cleaning [9].

Because *A*. *baumannii* is primarily transmitted by close contact with infected persons or with contaminated surfaces, surface contamination has been implicated in the transmission of *A*. *baumannii*. Nevertheless, it is difficult to eradicate the organisms from contaminated surfaces using conventional cleaning and disinfection methods [9, 10]. Therefore, other novel environmental disinfection methods are needed to help control the spread of multidrug resistant pathogens. For environmental cleaning, bacteriophages (phages) are a potential alternative strategy against CRAB strains. To date, it has been shown that several isolated phage strains can infect *A*. *baumannii* [11–13]. Our group isolated some specific phages for genomic analysis [14–16] and investigated the possible ways to apply phages for environmental cleaning[17]. Here, we used bacteriophages as an adjuvant disinfectant for environmental cleaning and evaluated the efficacy of a phage aerosol on CRAB nosocomial transmission in ICUs. This study was performed to determine whether a phage aerosol applied in ICUs would cause a significant impact on nosocomial incidence density, carbapenem-resistance rates and antimicrobial drug consumption amounts.

## Materials and Methods

### Setting and patients

This study was conducted in one medical center with 945-beds located in eastern Taiwan. It is the main tertiary referral center serving eastern Taiwan. In addition, this study was approved by the institutional review board of the Buddhist Tzu Chi General Hospital (IRB101-35). The ICU includes 20 beds for medical ICU (MICU) and 40 beds for surgical ICU (SICU). We retrospectively evaluated the infection control electronic database for new acquisitions of *A*. *baumannii* from January 1, 2012, to December 31, 2013. The study comprised two periods: period 1 (P1) occurred from January 1, 2012, to February 28, 2013 and was the baseline period, and period 2 (P2) was the following 8-month intervention period [from March 1, 2013, to December 2013]. The incidence density of carbapenem-resistant *Pseudomonas aeruginosa* (CRPA) was used as a control to evaluate the effect of bacteriophage on CRAB.

### *A*. *baumannii* isolates and identification

*A*. *baumannii* was identified by standard methods and confirmed using the VITEK-2 system (BioMerieux, Hazelwood, MO). Antimicrobial susceptibility tests were performed using the disk diffusion method and the VITEK-2 system as recommended by the 2006 Clinical and Laboratory Standards Institute. The minimal inhibitory concentration (MIC) of imipenem and meropenem was determined by the E-test (AB Biodisk, Solna, Sweden), and the resistant breakpoint of CRAB to imipenem and meropenem was defined as a MIC > 8 μg/mL.

### Active bacteriophage isolation and selection

In our previous study, we demonstrated the ability of the ϕAB2 phage to reduce multidrug-resistant *A*. *baumannii* (MDRAB) in suspension and on experimentally contaminated glass surfaces [17]. In the past 3 years, 24 active bacteriophage isolates against *A*. *baumannii* were successfully collected from sewage or river water by the phage research team in our hospital. Over 98% of CRAB isolates caused nosocomial infection in our ICUs and were sensitive to the panel of active phages. To simplify our experimental conditions and limit the possibility of CRAB developing phage resistance during our study period, we preferred to sequentially use different single phages rather than a combination of phages. When a CRAB patient was moved to the MICU or SICU, phage typing was performed for each CRAB strain that was clinically isolated from the patient. To select the optimal phage for aerosol decontamination, only the most active single phage for lysis was selected based on the score of the lysis zone of the target CRAB using the agar overlay method [18].

### Environmental decontamination

In this study, two isolation rooms were provided in each ICU and the isolation room had a volume of approximately 27 m^3^. Prior to the intervention (P1), routine terminal decontamination of the environment was performed using sodium hypochlorite (0.06%) for cleaning large surfaces, sinks, toilets, and fluid spills. An alcohol-based preparation (75%) for rapid disinfection of small surfaces was also used. During the intervention period (P2), the room was still decontaminated in advance using routine terminal cleaning processes. Then, the phage aerosol was generated for cleaning after the routine decontamination process. An ultrasonic humidifier (Mister 4, DDON LTD Inc., Taiwan) was used for 5 min to nebulize the bacteriophage stock into normal saline. The humidifier used a metal diaphragm that vibrated at an ultrasonic frequency to create phage droplets in the form of a cool fog. This created a fine mist with major droplets of approximately 5–7 μm in diameter that evaporate into the air flow and saturate a space of 27 m^3^ (3 × 3 × 3 m) within 2 and half min. The phage concentration of stock in the ultrasonic humidifier was 10^7^ PFU (plaque forming unit)/ml and approximately 500 ml was used in each experiment. In the room with a volume of 27 m^3^, the phage density that settled on the surface was calculated to be 5.5 × 10^4^ PFU/cm^2^ if we assume that generated phage aerosols all settled on the horizontal surfaces. Subsequently, patients with culture proven CRAB infection were then transferred to the isolation room when the phage aerosol cleaning had been completed. The bacteriophage decontamination program was conducted starting from March through December 2013.

### Data collection

The outcome of the study was the nosocomial incidence density of CRAB including new acquisitions or colonizations with CRAB among hospitalized patients in both the SICU and MICU. The nosocomial incidence density was defined as the isolation of the first CRAB isolate after 48 h of hospitalization in a patient without a previous history of *A*. *baumannii*. Multiple positive isolates from the same patient were included in the first isolated patient only. The rates were calculated in the SICU and MICU based on the number of new acquisitions per 1,000 patient-days. Our study used CRPA as a control to evaluate the efficacy of phage aerosol cleaning because the incidence density of other gram-negative bacteria, including carbapenem-resistant *Escherichia coli*, *Klebsiella pneumoniae*, *Proteus mirabilis*, and *Serratia marcescens*, were approximately zero in our institute. The second outcome was the mean carbapenem resistance rates to *Acinetobacter baumannii* in the ICU. The total isolates of CRAB were divided by the total isolates of *A*. *baumannii* and multiplied by 100. The third outcome was the antimicrobial consumption in both intensive care units and was calculated as the number of defined daily doses (DDD) per 1000 patient-days following the recommendation of the World Health Organization. We selected tigecycline, colistin methanesulfonate, meropenem and imipenem, which were used for the treatment of CRAB.

### Statistical analysis

The data were analyzed using a univariate Poisson regression (proc genmod) to generate an outcome variable (number of CRAB acquisitions) following a Poisson distribution. The rate of new acquisitions of CRAB per month was the dependent variable in the model, and the “phage intervention” served as the independent variable. We believe that CRAB acquisition is irrelevant to the month; therefore, the month variable was not considered in the Poisson model. This rate was calculated as the number of new CRAB cases detected in a month divided by the monthly hospital patient-days and multiplied by 1,000. The independent variable is based on whether the intervention procedure occurs. The natural log of the patient days per month was used as an offset in the Poisson regression to model the acquisition rates per 1,000 patient-days. The Mann-Whitney *U* test was used to compare the CRAB and CRPA incidence density between the two study periods and the antimicrobial consumption. All of the analyses were performed using SAS version 9.2 (SAS Institute Inc., Cary, NC).

## Results

### Number of isolates and rates of new acquisitions of CRAB and CRPA

**Fig 1:**
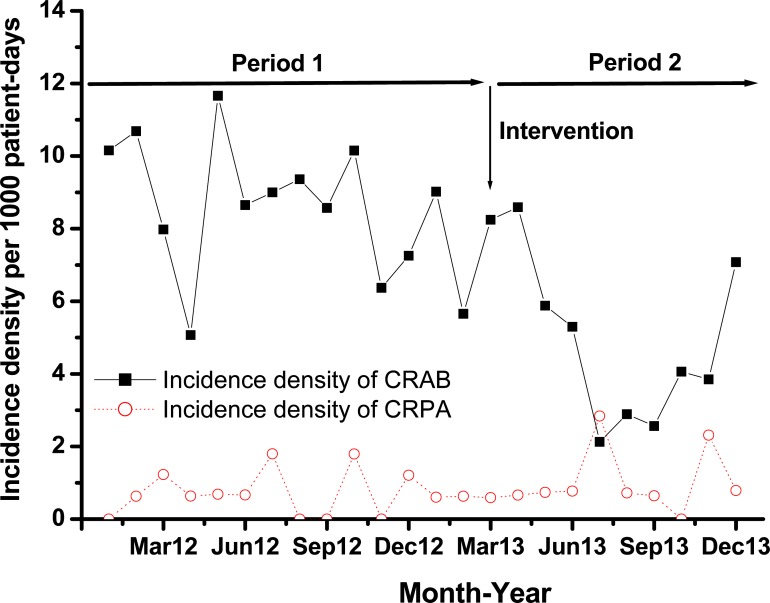
Using phage-containing aerosols as an intervention strategy to affect the trends in the incidence density of CRAB and CRPA per 1000 patient-days from 2012 to 2013. Prior to the intervention, routine decontamination was only performed using sodium hypochlorite. During the intervention period, the room was decontaminated by routine cleaning combined with a phage aerosol cleaning process.

**Fig 2:**
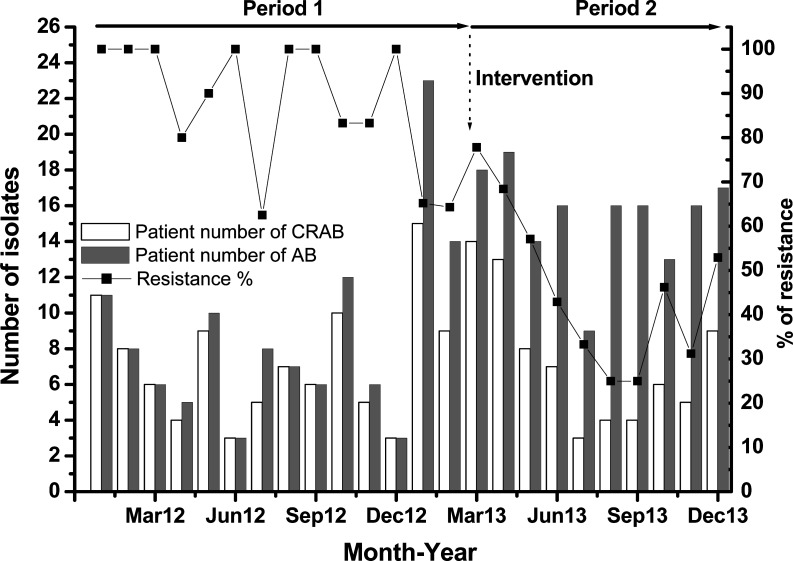
Using phage-containing aerosols as an intervention strategy to affect the trends in the patient number of CRAB or AB (left y-axis) and percent resistance (right y-axis) among *A*. *baumannii* in the ICUs from January 2012 to December 2013.

During period P2, 8 selected phages among our 24 active phage isolates were applied during aerosol cleaning (Table 1). These 8 selected phages were sequentially used for a total of 52 times based the phage typing results from each CRAB strain isolated from the patient. During the study, a total of 264 new acquisitions of CRAB were identified in the ICUs (191 and 73 in the P1 and P2, respectively). The rates of new acquisitions of CRAB for ICUs decreased from 8.57 per 1000 patient-days in the pre-intervention period to 5.11 per 1000 patient-days in the intervention period (*p* = 0.0029). No significant change in the rates of new acquisitions of CRPA for the ICUs were observed with a slight increase from 0.72 per 1000 patient-days in the pre-intervention period to 0.98 per 1000 patient-days in the intervention period (*p* = 0.3837) (Fig 1). In addition, the percentage of carbapenem resistant isolates with *A*. *baumannii* decreased from 87.76% to 46.07% in the ICUs (*p* = 0.001) (Fig 2). Otherwise, the percentage of carbapenem-sensitive *A*. *baumannii* (CSAB) increased from 17.21% to 52.60% in the ICUs (*p* = 0.001). In the P2 period, there was a 47.5% reduction in the rate of CRAB clinical culture; however, there was 67.3% increase in the rate of CSAB in comparison with the P1 period.

**Table 1:** The specific phage used for the cleaning of ICUs. Different single phages were sequentially used according to the phage typing results from each CRAB strain that was clinically isolated from the patient.

Podoviridae	Times used for cleaning*	Related reference^#^
ϕAB1	2	[16][17]
ϕAB2	9	[15][17][29]
ϕAB6	17	[17]
ϕAB7	3	[17]
ϕ4C08	3	-
ϕ8C07	1	-
Myoviridae		
ϕAB11	5	[17]
Siphovirdae		
ϕ5C05	12	-

* How many times each specific phage was used alone for cleaning.

^#^ The related reference for each specific phage.

### Antimicrobial drug consumption amounts

**Fig 3:**
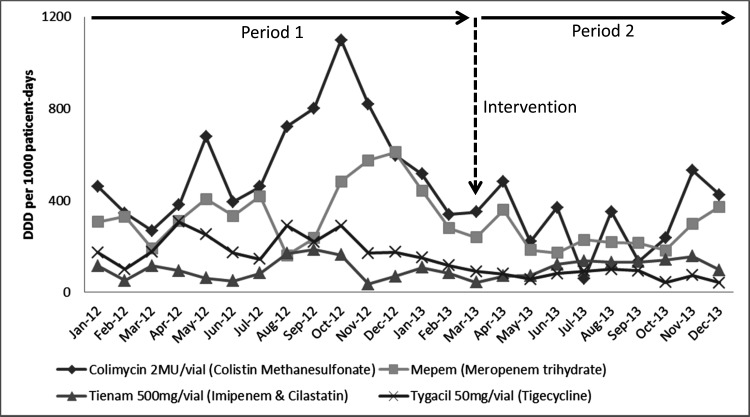
The use of phage-containing aerosols as an intervention strategy to affect the trends in the consumption of antimicrobial drugs in DDD per 1000 patient-days. The related antimicrobial drugs used for CRAB treatment are divided into four major groups: colimycin, mepem, tineam, and tygacil.

The consumption pattern of the antimicrobial use for the treatment of CRAB infection, including tigecycline, colistin methanesulfonate, meropenem and imipenem, are shown in Fig 3. All of the antimicrobials had a significant reduction in consumption except imipenem. In response to the intervention, colistin methanesulfonate use decreased from 7876 DDD per 1000 patient-days during P1 to 3158 DDD per 1000 patient-days in the P2 (*p* = 0.0177). Tigecycline decreased from 2737 DDD per 1000 patient-days in P1 to 753 DDD per 1000 patient-days in P2 (*p* = 0.0005). Meropenem decreased from 5084 DDD per 1000 patient-days in P1 to 2469 DDD per 1000 patient-days in P2 (*p* = 0.0385). Imipenem decreased from 1384 DDD per 1000 patient-days in P1 to 1101 DDD per 1000 patient-days in P2 (*p* = 0.3959).

## Discussion

It has been demonstrated that *A*. *baumannii* can survive for a long time on inanimate surfaces [19]; this contributes to the clonal spread of isolates and facilitates person-to-person transmission and environmental contamination. Rigorous infection control measures, including strict isolation, environmental cleaning, staff education, and proper hand hygiene, are important in controlling multidrug-resistant *A*. *baumannii* [20]. The above-mentioned infection control measures need to be implemented for a long time or closure of the hospital units is required to control the transmission of multidrug-resistant organisms [21]. Consequently, other novel environmental disinfection methods are needed to help control the spread of multidrug-resistant pathogens.

Phage is a natural parasite of bacteria; it was first discovered by Felix D’Herelle in 1917. Bacteriophages are often highly specific to a bacterial species and they are non-toxic to eukaryotic cells, including animals and plants, and usually increase in titer as they infect, multiply and kill their target microbes. Furthermore, they are readily isolated from a wide range of environments [22, 23]. However, mixed therapeutic results, a poor understanding of phage biology, and the advent of broad-spectrum antibiotics has led to the decline of phage therapy in the Western world [22, 24]. To date, phage products have been developed for the decontamination of food, plants, fields, and livestock and have been approved by FDA and are now in use [25–28]. However, to our knowledge, the use of aerosolized phage to reduce the levels of bacteria on hard surfaces has never been studied.

The first active phage shown to specifically infect MDRAB was characterized in 2010 [17]. In this study, the model phage ϕAB2 exhibited rapid adsorption (>99% adsorbed in 8 min), a short latent period (<10 min), and a large burst size [17]. Moreover, it was capable of infecting a wide spectrum of *A*. *baumannii* strains and caused complete lysis [17]. Previously, we demonstrated that the ϕAB2 phage had the potential to reduce MDRAB contamination in liquid suspensions or on hard surfaces [29]. The ϕAB2 phage at a concentration of at least 10^5^ PFU/mL on an *A*. *baumannii* M3237 suspension killed >99.9% of *A*. *baumannii* M3237 after 5 min, regardless of the *A*. *baumannii* M3237 concentration [29]. In addition, ϕAB2 was demonstrated to survive on a dried glass surface for 2 months and its infectivity retention was more than 50% when stored in deionized water after 360 days at 4°C [29]. Here, we used 8 phages, including ϕAB2, for environmental decontamination and the result was significant. The rates of new acquisitions of CRAB and the percent of resistant isolates of *A*. *baumannii* were decreased when these results were compared with that in the pre-intervention period. These results might be related to the fact that our phages that were applied for aerosol decontamination were customized by each CRAB strain isolated from the patient. Once the CRAB had been successfully killed by each customized phage, it could prevent the new acquisition of this CRAB strain for a period of time. At the same time, the percent of resistant isolates of *A*. *baumannii* were decreased. It is worth noting that the number of CSAB increased when the phage aerosol was applied. This could be related to the fact that our phage was only customized by CRAB strains; the applied phage cannot infect the CSAB strains because phages are highly specific to their host strains. Once the CRAB was decreased, the vacancy in the environment could be replaced by CSAB or other microbe species. The increasing number of non-drug resistant strains would make antibiotics a more effective therapy.

In addition, although we did not have specific antimicrobial consumption controls during the study, the overall consumption of the antimicrobial used during the P2 period of the study significantly decreased or remained the same (Imipenem). The relationship between the incidence density of CRAB and antimicrobial consumption may be difficult to evaluate, but previous studies also agreed that a successful infection control strategy may be related to decreased antimicrobial consumption [30, 31]. The reason that the consumption of imipenem did not decrease, but meropenem did, may be because meropenem is more commonly used as a combination treatment for CRAB, which is four times higher than imipenem use in our institute. The low consumption of imipenem might explain the insignificant change between period P1 and P2.

Recently, phages have been widely used for the decontamination of food, but limited data are available concerning the efficacy of phages in this decontamination application. Moreover, aerosolized phages applied in ICUs to prevent infections caused by CRAB have not been demonstrated. A study by Boyce et al. indicated that 39% of the sampled surfaces from the ICU environmental samples may have *A*. *baumannii*. Our recent study also demonstrated that the concentrations of CRAB varied widely from 0.0 to 201.8 CFU/cm^2^ on the surfaces of the common hospital environment before routine cleaning [32]. Even if the surfaces were cleaned and treated with a chlorine related compound, CRAB may still remain on these surfaces (0.0 to 0.6 CFU/cm^2^) [32]. To compensate for this deficiency, the application of phage-containing aerosols after routine cleaning for decontamination is time saving and the nooks and crannies of the surface may be easily cleaned during the process. The density of phage that settled on the surface may be sufficient against the remaining CRAB after routine cleaning. However, routine cleaning by chemical disinfectants is still necessary to decrease the microbe’s level in a specific and short time. Yet, in case of CRAB strains, the absence of these antibiotic-resistant species after routine cleaning can be defined as “real clean” [33]. A comprehensive decontamination method such as phage aerosol application that can be conducted after the routine cleaning process is recommended. However, we do not recommend using chemical disinfectants when the phage aerosol cleaning is finished because the disinfectants, such as sodium hypochlorite or alcohol, can inactivate the applied phage.

One limitation of this study is the subsequent lack of assessment for any development of phage resistance [26, 34]. We also did not evaluate the possibility that CRAB could develop resistance to our specific phages. However, because our different phages were sequentially and alternately used, this application would limit the emergence of phage resistance in CRAB for a period of time. For phage resistance development, Capperelli et al. found an average resistance frequency of 1.3 × 10^−8^ for *S*. *aureus* A170 treated with phages *in vitro* [23]. This result suggested that phage resistance may not be a very frequent event. In addition, Capperelli et al. were unable to isolate any phage-resistant *Staphylococcus aureus* strains from mice treated with phages. A second limitation is that the patient must vacate the room before and after aerosolization with the selected active phage. Some frequently touched areas, including patient beds and bedrails, were not decontaminated with phage. This may affect the results of the study. Further study is also required to validate whether repeated aerosolization with the phage is needed.

In conclusion, this is the first study to use a phage as an environmental biocontrol agent to decontaminate CRAB colonization in ICUs. Phage can significantly reduce the incidence of CRAB and provides adjuvant activity for the control of CRAB infection in healthcare settings.
